# Spinal toll like receptor 3 is involved in chronic pancreatitis-induced mechanical allodynia of rat

**DOI:** 10.1186/1744-8069-7-15

**Published:** 2011-02-22

**Authors:** Nian-Song Qian, Yong-Hui Liao, Quan-Xing Feng, Yu Tang, Ke-Feng Dou, Kai-Shan Tao

**Affiliations:** 1 Department of Hepatobiliary Surgery, Xijing Hospital, Fourth Military Medical University, Xi'an 710032, PR China; 2 Department of Hepatobiliary surgery, Chinese People's Liberation Army General Hospital, Beijing 100853, PR China; 3 Department of Breast surgery, Graduate School of Medicine, Kyoto University, Kyoto 606-8507, Japan; 4 Xijing Hospital of Digestive Diseases, Fourth Military Medical University, Xi'an 710032, PR China; 5 Department of Ultrasound, Chinese People's Liberation Army 302 Hospital, Beijing 100039, PR China

## Abstract

**Background:**

Mechanisms underlying pain in chronic pancreatitis (CP) are incompletely understood. Our previous data showed that astrocytes were actively involved. However, it was unclear how astrocytic activation was induced in CP conditions. In the present study, we hypothesized that toll-like receptors (TLRs) were involved in astrocytic activation and pain behavior in CP-induced pain.

**Results:**

To test our hypothesis, we first investigated the changes of TLR2-4 in the rat CP model induced by intrapancreatic infusion of trinitrobenzene sulfonic acid (TNBS). Western blot showed that after TNBS infusion, TLR3, but not TLR2 or TLR4, was increased gradually and maintained at a very high level for up to 5 w, which correlated with the changing course of mechanical allodynia. Double immunostaining suggested that TLR3 was highly expressed on astrocytes. Infusion with TLR3 antisense oligodeoxynucleotide (ASO) dose-dependently attenuated CP-induced allodynia. CP-induced astrocytic activation in the spinal cord was also significantly suppressed by TLR3 ASO. Furthermore, real-time PCR showed that IL-1β, TNF-α, IL-6 and monocyte chemotactic protein-1 (MCP-1) were significantly increased in spinal cord of pancreatic rats. In addition, TLR3 ASO significantly attenuated CP-induced up-regulation of IL-1β and MCP-1.

**Conclusions:**

These results suggest a probable "TLR3-astrocytes-IL-1β/MCP-1" pathway as a positive feedback loop in the spinal dorsal horn in CP conditions. TLR3-mediated neuroimmune interactions could be new targets for treating persistent pain in CP patients.

## Background

Chronic pancreatitis (CP) is a severe inflammatory and painful disease of the exocrine pancreas. Constant, recurrent, and serious abdominal pain is one of the most common symptoms in CP, present in 80-90% of the patients [1]. However, the pain mechanisms in CP are incompletely understood and probably are multifactorial, including pancreatic and extrapancreatic causes [2]. Experimental human pain studies show that pain processing in the central nervous system (CNS) is abnormal in CP-related neuropathic pain disorders [3,4]. A recent study showed that in the patients of CP and pancreatic cancer, pancreatic neuropathy could bring "neural remodeling" and alter pancreatic innervation [5]. These results highly suggest that neuroplastic changes in the CNS are probably important contributors to the CP-induced chronic pain. And it has been reported that pain in CP shares many characteristics of neuropathic pain [6-8].

Neuron-immune interactions and neuron-glial crosstalk in the spinal dorsal horn play a pivotal role in neuroplastic changes and neuropathic pain [9,10]. The involvement of neuroimmune interactions in CP-induced pain has also been reported [2]. Our recent study showed that astrocytes were activated in the thoracic spinal cord in a rat model of CP induced by intrapancreatic infusion of trinitrobenzene sulfonic acid (TNBS), and inhibiting astrocytic activation could attenuate pain of CP [11]. We thus estimated that, in CP conditions, astrocytes might be activated through some receptors, and then produced signaling molecules that could further enhance neuronal activity, contributing to pain facilitation [12]. However, it is still unclear which receptor(s) mediated astrocytic activation in CP conditions.

Spinal Toll-like receptors (TLRs) play a key role in neuron-immune interactions and neuron-glial crosstalk in chronic pain conditions [13-15]. TLR2-4 have been clarified to be major mediators in neuropathic pain [14,16,17]. In general, in response to stimulation by endogenous and exogenous signals, TLRs could induce glial activation in which multiple TLRs could trigger and tailor innate immune responses of glia by altering production of pain-associated pro-inflammatory cytokines/chemokines [15]. However, there is still no report on the contribution of TLRs in CP related pain. Interestingly, TLRs have been implicated in the process of pancreatitis [18]. A recent study showed that intraperitoneal injection of TLR3 activator could successfully induce CP-like pathological changes [19].

In the present study, we hypothesized that TLRs (TLR2-4) were involved in astrocytic activation and pain behavior in the process of CP-induced pain. To test our hypothesis, we first investigated the expression changes of TLR2-4 following TNBS-induced CP. We found that TLR3, but not TLR2 or 4, was increased in the thoracic spinal dorsal horn in the process of CP. Then we detected the cellular localization of TLR3 with double immunostaining and observed that TLR3 was highly expressed on spinal astrocytes. We further used a kind of TLR3 antisense oligodeoxynucleotide (ASO) to decrease the expression of TLR3 and observed the behavioral and biochemical changes in the spinal cord.

## Results

### TNBS infusion induced CP and mechanical allodynia

**Figure 1:**
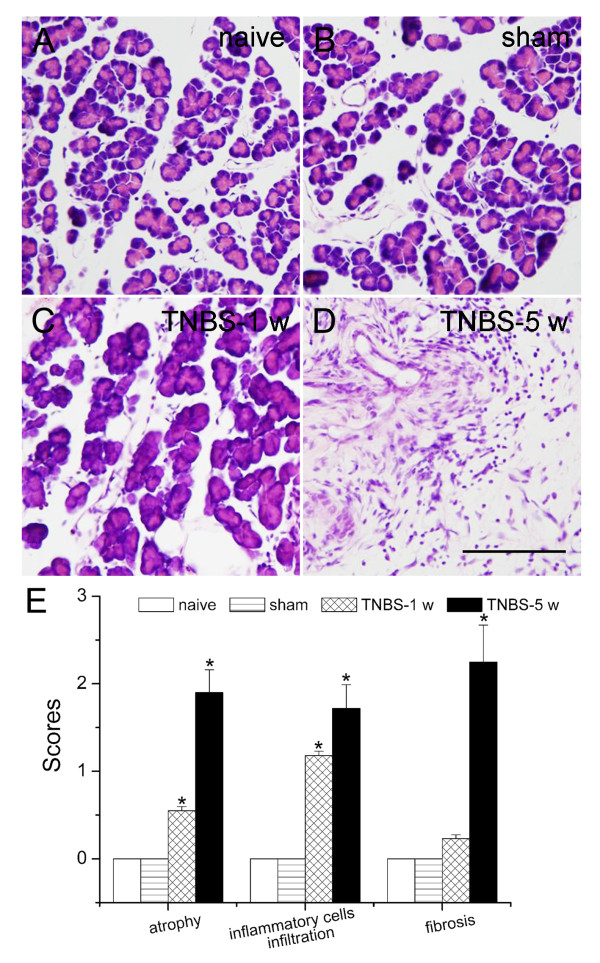
**Trinitrobenzene sulfonic acid (TNBS) infusion-induced rat chronic pancreatitis**. (A-D) Hematoxylin and eosin staining of the pancreas in (A) naïve group, (B) sham operated group, (C) trinitrobenzene sulfonic acid (TNBS)-1 w group and (D) TNBS-5 w group. (E) The severity of CP was morphological assessed by semiquantitative scoring: graded glandular atrophy (0-3); intralobular, interlobular and periductal fibrosis (0-3); inflammatory cells infiltrations (0-3). * *P *< 0.05 compared with that of naïve group.

**Figure 2:**
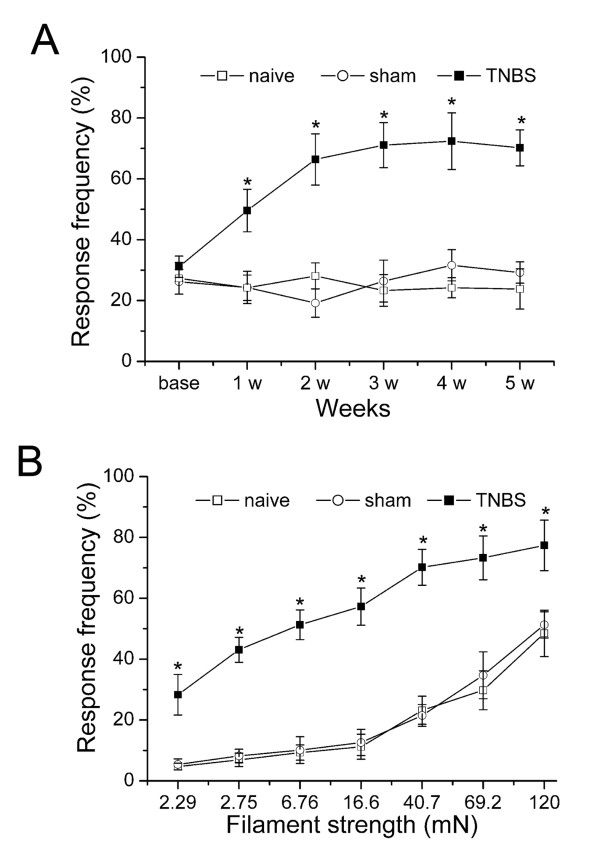
**Chronic pancreatitis-induced rat mechanical sensitivity of the abdomen**. (A) Response frequencies to mechanical stimulation of the abdomen with the 40.7 mN of von-Frey filaments before (base) and up to 5 w after pancreatic infusion with TNBS (TNBS group, n = 6), vehicle (sham group n = 6) or naïve control (n = 6). (B) Response frequencies to mechanical stimulation of the abdomen with von-Frey filaments of various strengths at 5 w in the rats of TNBS (n = 6), sham (n = 6) or naïve (n = 6) groups. * *P *< 0.05 compared with that of naïve group.

In the naïve and sham rats, the pancreas presented a normal appearance. While in 5 w after TNBS infusion, the pancreas showed significant acinar atrophy, inflammatory infiltration, and periductular and intralobular fibrosis, stromal proliferation (Figure 1). CP-induced persistent mechanical allodynia is characterized by increase of abdomen response frequencies (RFs) [4,7]. We observed that rats with CP showed persistent mechanical hypersensitivity in the abdomen. The mechanical allodynia was evident 1 w (RFs = 49.6 ± 7.5% for TNBS group, *P *< 0.05, *vs *naive group; Figure 2A) after TNBS infusion and persistent up to 5 w (RFs = 70.2 ± 5.9% for TNBS group, *P *< 0.05, *vs *naive group). There was no significant difference between sham and naive group at any time points. Then, we examined the effects of different stimulations on RFs of rats with von-Frey filaments of various strengths from 2.29 to 120 mN on 5 w post CP induction. We observed that at 5 w after TNBS infusion, RFs of rats were significantly higher at all filaments tested compared to either sham or to naive rats (*P *< 0.05, Figure 2B). Thus we confirmed that intrapancreatic infusion of TNBS produced CP and increased the sensitivity to mechanical probes in the abdomen.

### CP significantly up-regulated TLR3 expression in the thoracic spinal dorsal horn

**Figure 3:**
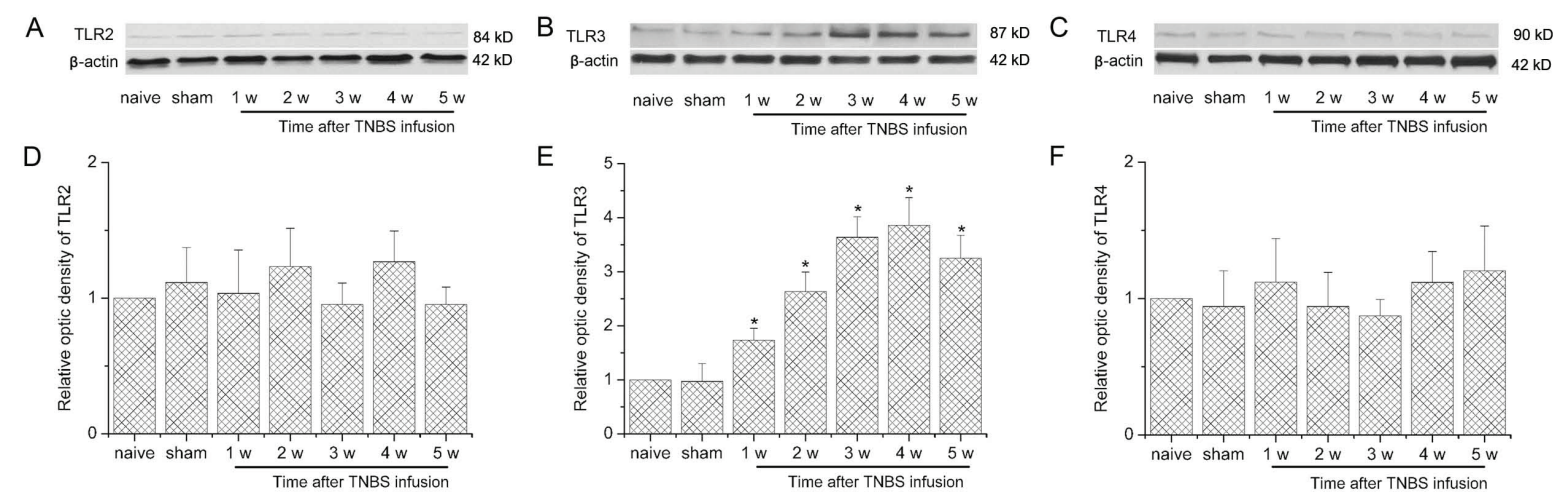
**Chronic pancreatitis-induced significant increase of toll like receptor (TLR) 3 in the thoracic spinal dorsal horn**. (A-C) Representative Western blot data showing the expressions of TLR2-4 in naïve, sham and 1-5 w following TNBS infusion. (D-F) statistical analysis on Western blot results. * *P *< 0.05 compared with that of naïve group. In naïve or sham operated rats, TLR 2-4 expressions in the thoracic spinal dorsal horn were at very low levels. After intrapancreatic infusion of TNBS, TLR 2 and TLR 4 were still at a very low level, compared with that of naïve and sham group. TLR 3 was significantly increased in the spinal cord, from 1 w after CP induction. TLR 3 was increased gradually and maintained at a very high level up to 5 w.

**Figure 4:**
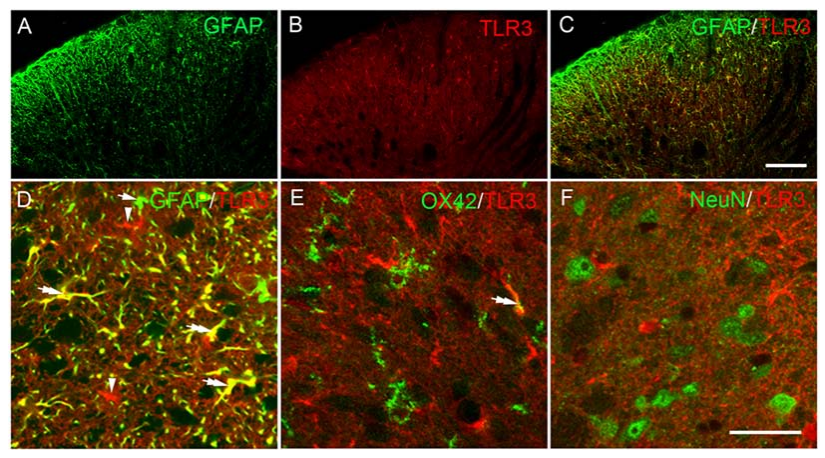
**Double immunostaining showing the cellular location of toll like receptor (TLR) 3 with antibodies against astrocytic specific marker glial fibrillary acidic protein (GFAP, A-D), microglial marker OX42 (E) and neuronal marker NeuN (F)**. We observed that TLR 3 was highly expressed on spinal astrocytes, and also expressed on microglia in a very low level. Single arrows: GFAP-positive and TLR 3-negative cell structures; Single arrowheads: GFAP-negative and TLR 3-positive cell structures; Double arrows: double-labeled cell structures. Scale bars = 200 μm in C, applied in A-C, 50 μm in F, applied in D-F.

We then sacrificed the rats at different time points and observed the changes of TLR2-4 expressions. Western blot analysis indicated that in naïve or sham operated rats, TLR2-4 expressions in the thoracic spinal dorsal horn were very low (Figure 3A, C). After intrapancreatic infusion of TNBS, TLR2 and TLR4 expressions were still very low, compared with that of naïve or sham group (Figure 3D, F; *P *> 0.05). Interestingly, TLR3 was significantly increased in the spinal cord, from 1 w after CP induction (*P *< 0.05, Figure 3B, E) and was maintained at a very high level up to 5 w (*P *< 0.05, *vs *naive group). We previously observed a similar changing course of spinal astrocytic activation after CP induction [11], so TLR3 was assumed to be expressed on spinal astrocytes and mediated CP-induced astrocytic activation. We thus observed the cellular localization of TLR3 in the thoracic spinal dorsal horn 5 w after CP induction. Immunofluorescence double labeling showed that TLR3 was highly expressed on glial fibrillary acidic protein (GFAP)-positive astrocytes (Figure 4A-D), with a very low level on spinal microglia (Figure 4E). No obvious TLR3 immunoreactivities could be detected on spinal neurons (Figure 4F).

### Intrathecal infusion of TLR3 ASO significantly attenuated CP-induced mechanical allodynia

**Figure 5:**
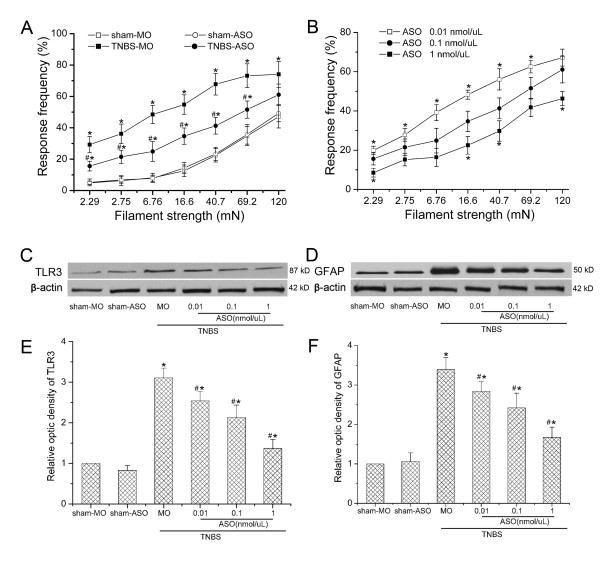
**Intrathecal infusion of toll like receptor (TLR) 3 antisense oligodeoxynucleotide (ASO) on mechanical allodynia and astrocytic activation**. TLR 3 ASO or mismatch oligodeoxynucleotide (MO) was intrathecally infused for 1 w after pancreatitis induction. (A) Response frequencies to mechanical stimulation of the abdomen with von-Frey filaments of various strengths at 5 w in the rats of trinitrobenzene sulfonic acid (TNBS)-MO, sham-MO, sham-ASO (0.1 nmol/μL) and TNBS-ASO (0.1 nmol/μL) group. *, # *P *< 0.05 compared with that of sham-MO or TNBS-MO group, respectively. (B) TNBS-induced allodynia was significantly attenuated by TLR 3 ASO in a dose-dependent manner. * *P *< 0.05 compared to 0.1 nmol/μL group. (C, D) Representative Western blot data showing the expressions of TLR 3 and glial fibrillary acidic protein (GFAP) following different treatments. (E, F) statistical analysis on Western blot results. Intrathecal infusion of TLR 3 ASO, but not TLR 3 MO, significantly blocked CP-induced TLR 3 up-regulation and astrocytic activation. *, # *P *< 0.05 compared to sham-MO or TNBS-MO group, respectively.

In order to testify our hypothesis that TLR3 contributed to CP-induced neuropathic pain, we used a kind of TLR3 ASO to knockdown the expression of TLR3 and observed the behavioral consequences as well as cellular and molecular changes. TLR3 ASO could specifically bind to the TLR3 RNA and reduce spinal TLR3 expression. TLR3 mismatch oligodeoxynucleotide (MO) was selected as a negative control. Infusion with TLR3 ASO (0.1 nmol/μl, from 4th w to 5th w following TNBS infusion) significantly, even though not completely, attenuated mechanical allodynia (*P *< 0.05, Figure 5A). However, TLR3 ASO did not influence RFs of sham operated rats. We further observed that TNBS-induced allodynia was remarkably attenuated by TLR3 ASO, in a dose-dependent manner (Figure 5B). Western blot confirmed that intrathecal infusion of TLR3 ASO, but not TLR3 MO, significantly blocked CP-induced TLR3 up-regulation (Figure 5C). These results suggested that TLR3 might contribute to CP-induced mechanical allodynia.

### TLR3 ASO significantly reversed CP-induced astrocytic activation, as well as cytokines expressions

**Figure 6:**
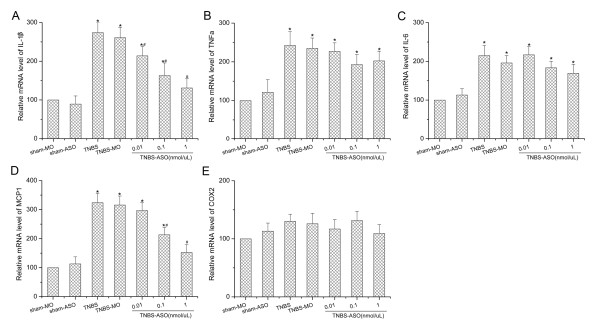
**Intrathecal infusion of toll like receptor (TLR) 3 antisense oligodeoxynucleotide (ASO) on cytokine/chemokine expressions revealed by real-time PCR**. Changes of (A) IL-1β, (B) TNF-α, (C) IL-6, (D) monocyte chemotactic protein-1 (MCP-1), and (E) cyclooxygenase 2 (COX-2) mRNA levels were determined in the T10 region of the dorsal spinal cord of pancreatic or sham rats. Significant increases of IL-1β, TNF-α, IL-6 and MCP-1, except COX-2, were observed in spinal cord of pancreatic rats. TLR3 ASO significantly attenuated up-regulation of IL-1β and MCP-1, but not TNF-α or IL-6. *, # *P *< 0.05 compared to sham-MO or TNBS-MO group, respectively.

Since TLR3 was highly expressed on spinal astrocytes after CP induction, we thus investigated the role of TLR3 ASO on CP-induced astrocytic activation. CP-induced astrocytic activation in the thoracic spinal dorsal horn was remarkably suppressed by TLR3 ASO (Figure 5D). GFAP expression in the TNBS-MO group was significantly higher than that of sham group (*P *< 0.05). However, GFAP levels in the TNBS-ASO groups were much lower than that of the TNBS-MO group, although still higher than that of the sham groups. We then further measured the cytokines expression in the rat thoracic spinal dorsal horn following different treatments. A significant up-regulation of cytokines was observed after CP-induced chronic pain. In the TNBS-MO group, we observed significant increases of IL-1β (261.4% ± 26.6, *P *< 0.05, Figure 6A), TNF-α (235.1% ± 25.3, *P *< 0.05, Figure 6B), IL-6 (195.7% ± 18.9, *P *< 0.05, Figure 6C) and monocyte chemotactic protein-1 (MCP-1) (315.8% ± 30.3, *P *< 0.05, Figure 6D) compared with those of sham groups. Intrathecal infusion of TLR 3 ASO could significantly attenuated CP-induced up-regulation of IL-1β and MCP-1, in a dose dependent manner. However, TNF-α or IL-6 was not significantly influenced by TLR3 ASO. However, cyclooxygenase-2 (COX-2) in the thoracic spinal dorsal was not increased in CP conditions, either influenced by intrathecal infusion of TLR3 ASO (Figure 6E).

## Discussion

Cumulating evidence supports that the inflammatory response in the spinal cord plays an important role in inducing and maintaining pathological pain [20-23]. In this context, we recently demonstrated that in a rat model of CP, astrocytic activation could be observed in the thoracic spinal dorsal horn [11]. In the present study, we further elucidate the molecular mechanisms underlying inflammatory process in the CNS in CP related pain states. We demonstrate that TLR3, but not TLR2 or TLR4, is increased in the spinal dorsal horn in a rat model of TNBS-induced CP. We further show that intrathecal injection of TLR3 ASO could significantly attenuate CP-induced mechanical allodynia, astrocytic activation, and cytokines expressions.

TLR2-4 have been implicated in pathological pain [13,14,16,17]. Previous studies showed a significant up-regulation of TLR2-4 in nerve injury-induced neuropathic pain models [14,17]. TLRs-deficient mice display significantly attenuated behavioral hypersensitivity and decreased expression of spinal glial activation and proinflammatory cytokines [24]. However, we observed no change of spinal TLR2 or 4 in CP model. We speculate the most probable reason is the different cellular localizations of TLR2, 3 and 4. Microglia constitutively express a wide range of TLR2-4 at high levels [25,26]. In comparison, astrocytes express TLR2 and 4 at lower levels, with particularly high levels of TLR3 [27-29]. This was also confirmed by double immunostaining in the present study. We reported that astrocytes, but not microglia, were activated in the spinal cord in CP conditions [11]. Probably, receptors on astrocytes, rather than on microglia, play more essential roles in pain of CP. In nerve injury-induced neuropathic pain model, astrocytes contribute more to the maintenance of mechanic allodynia, while microglia contribute more to the development [30,31]. In our previous study, no obvious spinal microglial activation in CP conditions was detected, possibly because of the observing window. However, we can not exclude the contribution of microglia in the initiation of CP-induced pain. In addition, the role of TLR2 and 4 in the very early stage (<1 w) of CP-induced pain remains to be elucidated. However, our results at least indicate that TLR3 is more important in the chronic phase of CP-induced pain.

We then further confirm that TLR3 is essential for astrocytic activation and mechanical allodynia. Antisense strategies have been widely used to locally knock-down a specific gene and protein, especially when specific inhibitors or antagonists are lacking. Previous study has also confirmed the role of TLR3 in spinal nerve injury induced pathological pain, with a kind of TLR3 ASO [16]. However, knock-down of TLR3 attenuates the activation of spinal microglia, but not astrocytes in the nerve injury model. We think the most probable reason for the different results is the difference of the models. Although somatic and visceral pain share some similarities in the underlying mechanism, there are still lots of differences [32]. Another explanation is the observing window. They observed in a very short period following nerve injury (1 w). Microglial responses typically precede astrocyte activation [31]. TLR3 activation in microglia might have a role in the early establishment of neuropathic pain. However, in the late stage, astrocytic TLR3 may contribute to the maintenance of neuropathic pain, as what we observed in the present study.

The coming question is how TLR 3 mediates astrocytic activation in CP conditions. The cytoplasmic portion of TLRs shows high similarity to that of the IL-1 receptor family, and is called the Toll/IL-1 receptor (TIR) domain [33]. In the signaling pathway downstream of the TIR domain, there is a TIR domain-containing adaptor, MyD88, which recruits IL-1 receptor-associated kinase (IRAK) to TLRs through interaction of the death domains of both molecules. Activation of IRAK leads to the activation of c-Jun N-terminal kinase (JNK) and NF-κB [34]. In neuropathic pain, activation of JNK has been proved to be exclusively present on spinal astrocytes and is essential for astrocytic activation and pain processing [12]. So probably in CP conditions, TLR3 could induce astrocytic JNK activation and consequent pain behavior. Traditionally, TLR3 is known to recognize double-stranded RNA [35] and double-stranded mRNA [36], following viral infection. In the process of CP, virus is considered as a critical factor [18]. A recent study even suggested double-stranded RNA, a ligand of TLR3 could induce CP [19]. However, in the TNBS-induced CP model, there is no viral infection. It is reported that the pro-inflammatory cytokines induced following CP could mediate TLR3 activation. Cultured astrocytes from human subcortical samples generally express TLR3 at very low levels. Upon activation with different pro-inflammatory cytokines (IL-1β, TNF-α etc), strong and preferential induction of TLR3 was observed consistently [37]. We indeed observed IL-1β, TNF-α and IL-6 were significantly increased in the spinal cord of pancreatic rats. Spinal IL-1β released following TNBS injection may also activate TLR3 because of the high similarity between TLR3 and IL-1 receptor. Pancreatic inflammation results in immune activation and release of a host of other pro-inflammatory cytokines [38,39], which could have direct influences on spinal TLR3. Alternatively, such substances may serve as retrogradely transported signals to influence gene activation in dorsal root ganglia (DRG) neurons, leading to synthesis of cytokines and their release in the dorsal horn termination area of the involved sensory nerves.

Pro-inflammatory cytokines could not only increase TLR3 expression, but could also be induced by TLR3 activation [40]. Activated astrocytes release varieties of cytokines/chemokines that enhance neuronal activity and further stimulate TLR3. In the present study, we observed that IL-1β and MCP-1 were increased following CP. Besides, TLR3 ASO could block IL-1β and MCP-1 expressions. These data suggest that IL-1β and MCP-1 are probably located on spinal astrocytes and are induced by TLR3 activation. After activation, astrocytes release IL-1β, which activates IL-1 receptor on neurons and consequently, enhancing neuronal activity and synaptic transmission [41,42]. Spinal astrocytes also release the chemokine MCP-1, which could induce pain behavior and phosphorylation of extracellular signal-regulated kinase (ERK) in superficial spinal cord dorsal horn neurons [12]. This positive feedback circuit enlarges the effect of nerve injury on nociception and makes it more difficult to develop a clinical therapy for pain of CP [10,43]. However, previous study shows that in cultured human astrocytes, TLR3 triggers enhanced production of anti-inflammatory cytokines including IL-9, IL-10, and IL-11, but not inflammatory cytokines observed in the present study. The possible explanation for the discrepancy could be different conditions (*in vitro *and *in vivo*) and different origins of astrocytes (brain and spinal cord).

TNF-α and IL-6 are increased in the spinal cord in CP conditions. However, TLR3 ASO has no effect on TNF-α or IL-6 expression. We thus conclude that TNF-α and IL-6 may not be induced by TLR3 activation, or synthesized in astrocytes. The possible origins of TNF-α and IL-6 are activated neurons in the spinal dorsal horn [23,44], or even macrophages. Besides, we did not observe expression change of spinal COX-2 following CP induction. However, other studies reported that TLR3 stimulation could induce TNF-α, IL-6 and even COX-2 *in vitro *[16,37,40]. Although recent studies suggest that COX-2 plays an essential role in peripheral and central pain processing [45], spinal COX-2 may not be involved in CP-induced pain. In addition, in the present study, we only detected spinal COX-2 expression 5 w after TNBS infusion. Whether spinal COX-2 is increased in the very early stages following CP-injection is to be determined.

## Conclusions

Our results provide evidence for the involvement of spinal TLR3 in CP-induced chronic pain. And we present a probable "TLR3 - astrocytes - IL-1β/MCP-1" pathway as a positive feedback loop in the spinal dorsal horn in CP conditions, which could be new targets for treating severe and persistent pain in CP patients.

## Methods

All experimental procedures received approval from the Animal Use and Care Committee for Research and Education of the Fourth Military Medical University (Xi'an, P. R. China) and also the ethical guidelines to investigate experimental pain in conscious animals. All efforts were made to minimize the number of animals used and their suffering.

### Induction of pancreatitis

Male *Sprague-Dawley *rats (250 and 300 g) were used in the study. Animals were given free access to drinking water and standard food pellets until 12 h prior to induction of pancreatitis, at which point food was withdrawn. TNBS-induced rat pancreatitis model was built according to our previous study [11]. Briefly, the common bile duct was closed temporarily near the liver with a small vascular clamp. A blunt 28 gauge needle with PE 10 tubing attached was inserted into the duodenum and was guided through the papilla into the duct and was secured with suture. TNBS solution (0.5 ml, 2%) in 10% ethanol in phosphate buffered saline (PBS, pH 7.4) was infused into the pancreatic duct over a period of 2-5 min at a pressure of 50 mmHg. After 30 min exposure to TNBS, needle and tubing were removed. The hole in the duodenum was sutured and the vascular clamp was removed restoring the bile flow. All the procedures in the sham group were same as that in the TNBS group, except that the same volume of saline instead of TNBS was infused into the duct.

### Administration of drugs

TLR3 ASO (5'-AACAATTGCTTCAAGTCCTCAAGTCC-3') and TLR3 MO (5'-ACTTCAACAGTAGACTACTAGACTAC-3') were synthesized by Sangon Biotechnology Co. (Shanghai, China). For intrathecal infusion, laminectomy was performed at the level of the thoracic vertebrae, under pentobarbital anesthesia (45 mg kg^-1^, *i.p.*). A polyethylene (PE10) catheter (I.D. 0.28 mm and O.D. 0.61 mm) was passed caudally from the T9 to the T12 level of the spinal cord, and 2 cm of the free ending was left exposed in the upper thoracic region. Rats were allowed to recover for a period of 3 - 5 days before further use. Only the animals judged as neurologically normal and that showed complete paralysis of the tail and bilateral hind legs after administration of 2% lidocaine (10 μL) through the intrathecal catheter were used for the following experiments. The catheter was connected to an osmotic pump (7-day pump, 1 μl/h; Durect, Cupertino, CA, USA) was filled with ASO (0.01, 0.1 or 1 nmol/μL) or MO (1 nmol/lL) in normal saline.

All the rats were divided into three groups: TNBS group (n = 54), sham group (n = 42), and naïve controls (n = 6). In order to study the time course of TLRs change, we sacrificed every 6 rats in TNBS group at 1, 2, 3, 4 and 5 w after TNBS infusion. At 4 w after surgery, other rats in TNBS (n = 24) and sham group (n = 24) were each further divided for drug injection: TNBS-ASO (or sham-ASO) group: TLR 3 ASO was intrathecally infused on pancreatitic (0.01, 0.1 or 1 nmol/μL, n = 6 each) or sham operated rats (n = 6, 1 nmol/μL only); TNBS-MO (or sham-MO) group: TLR3 MO was injected on pancreatitic (n = 6) or sham operated rats (n = 6). At 5 w after surgery, all the rats including naïve rats (n = 6) and sham rats without intrathecal injection (n = 6) were sacrificed for further experiments.

### Pain behavioral test

Mechanical allodynia was measured with von Frey filaments (Stoelting, Kiel, WI, USA). Testing was performed according to our protocol reported previously [11]. Prior to test, the belly skin of the rat was shaved and area designated for stimulation was marked. Rats were placed in a plastic cage with a mesh floor and were given 30 min for adaptation before testing. The von Frey filaments were applied from underneath through the mesh floor, in ascending order to the abdominal area at different points on the surface. A single trial consisted of 10 applications each for 1-2 s with a 15 s interval between applications allow the animal to cease any response and return to a relatively inactive position. A positive response consisted of the rat raising its belly (withdrawal response). The data were expressed as a percentage of the positive responses with each filament for each rat. In the time-course study, behavioral tests were performed before and once weekly for up to 5 w after induction of pancreatitis with a single force of von Frey filament (40.7 mN). In the strength-response study, rats were tested 5 w after pancreatitis induction with a series of von Frey filaments (2.29, 2.75, 6.76, 16.6, 40.7, 69.2 and 120 mN).

### Pancreatic histology

Rats were deeply anesthetized with sodium pentobarbital (60 mg/kg, *i.p.*) and the pancreas was obtained and then fixed in 4% paraformaldehyde in phosphate buffered (PB, pH 7.4) at 4°C overnight. Pancreatic tissue was then transferred to progressive xylene washes and was placed in cassettes and embedded in paraffin. Paraffin blocks were cut in 5-μm sections and stained with hematoxylin and eosin. Histological sections were analyzed by a pathologist in a double blinded manner. The severity of CP was morphologically assessed by semiquantitative scores according to previous reports: graded glandular atrophy (0-3); intralobular, interlobular and periductal fibrosis (0-3); inflammatory cells infiltrations (0-3) [46,47].

### Western blot

All animals were rapidly sacrificed and the thoracic (T) 10 spinal cord was rapidly harvested and then was frozen on the dry ice. Then the spinal dorsal horn was quickly micro-dissected. Spinal cord was then homogenized with a hand-held pestle in SDS sample buffer (10 ml/mg tissue), which contained a cocktail of proteinase inhibitors. After protein concentration was measured, proteins (50 μg) were heated for at 100°C for 5 min and loaded onto 10% SDS-polyacrylamide gels with standard Laemmli solutions (Bio-Rad Laboratories, CA, USA). The proteins were electroblotted onto a polyvinylidene difluoride membrane (PVDF, Immobilon-P, Millipore, Billerica, MA, USA). The membranes were placed in a blocking solution containing Tris-buffered saline with 0.02% Tween (TBS-T) and 5% non-fat dry milk, for 1 h, and incubated overnight under gentle agitation with primary antibodies: rabbit anti-TLR2 (1: 100; Santa-Cruz Biotechnology, Santa Cruz, California, USA), rabbit anti-TLR3 (1: 100; Santa-Cruz Biotechnology), rabbit anti-TLR4 (1: 100; Santa-Cruz Biotechnology) and rabbit anti-β-actin (1: 1000; Sigma, St. Louis, MO, USA). Bound primary antibodies were detected with the anti-rabbit horseradish peroxidase (HRP)-conjugated secondary antibody (1:10,000; Amersham Pharmacia Biotech Inc., Piscataway, NJ, USA). Between each step, the immunoblots were rinsed with TBS-T. All reactions were detected by the enhanced chemiluminescence (ECL) detection method (Amersham). The densities of protein blots were analyzed by using Labworks Software (Ultra-Violet Products, UK). The densities of target proteins and β-actin immunoreactive bands were quantified with background subtraction. The same size of square was drawn around each band to measure the density and the background near that band was subtracted. Target protein levels were normalized against β-actin levels and expressed as relative fold changes compared to the naïve control or to the sham-MO group.

### Real-time RT-PCR

Rats were deeply anesthetized with sodium pentobarbital (60 mg/kg, *i.p.*), the animals were rapidly sacrificed and the thoracic (T) 10 spinal cord was rapidly harvested and then was frozen on the dry ice. Then the spinal dorsal horn was quickly micro-dissected. RNA was extracted with Trizol (GIBCO/BRL Life Technologies Inc., Grand Island, NY, USA). Complementary DNA (cDNA) was synthesized with oligo (dT)_12-18 _using Superscript™ III Reverse Transcriptase for RT-PCR (Invitrogen, Carlsbad, CA, USA). The primers used in the present study were presented in Table 1. Equal amounts of RNA (1 μg) were used to prepare cDNA using the SYBR^® ^Premix Ex Taq™ (Takara, Tokyo, Japan) and analyzed by real-time PCR in a detection system (Applied Biosystems, Foster City, CA, USA). The amplification protocol was: 3 min at 95°C, followed by 45 cycles of 10 s at 95°C for denaturation and 45 s at 60°C for annealing and extension. All experiments were repeated twice and, in each experiment, PCR reactions were done in triplicate. Target cDNA quantities were estimated from the threshold amplification cycle number (C_t_) using Sequence Detection System software (Applied Biosystems). GAPDH was served as an endogenous internal standard control for variations in RT-PCR efficiency.

**Table 1:** Primers sequence for the rat genes characterized in this experiment

Genes	Primers		Accession number
MCP-1	Forward primer	5'-CAGATCTCTCTTCCTCCACCACTAT-3'	M57441
	Reverse primer	5'-CAGGCAGCAACTGTGAACAAC-3'	
IL-1β	Forward primer	5'-TGCTGATGTACCAGTTGGGG-3'	NM031512
	Reverse primer	5'-CTCCATGAGCTTTGTACAAG-3'	
IL-6	Forward primer	5'-GCCCTTCAGGAACAGCTATG-3'	NM012589
	Reverse primer	5'-CAGAATTGCCATTGCACAAC-3	
TNF-α	Forward primer	5'-TGATCGGTCCCAACAAGG A-3'	AY427675
	Reverse primer	5'-TGCTTG GTG GTTTGCTACGA-3'	
COX-2	Forward primer	5'-CAGTATCAGAACCGCATTGCC-3'	U03389
	Reverse primer	5'-GAGCAAGTCCGTGTTCAAGGA-3'	
GAPDH	Forward primer	5'-CCCCCAATGTATCCGTTGTG-3'	NM01008
	Reverse primer	5'-TAGCCCAGGATGCCCTTTAGT-3'	

### Immunofluorescent double labeling

At 5 w after TNBS infusion, rats were perfused through the ascending aorta with 100 ml of normal saline followed by 500 ml of 0.1 M phosphate buffer (PB, pH 7.3) containing 4% paraformaldehyde and 2% picric acid, under deep anesthesia with sodium pentobarbital (60 mg/kg, *i.p.*). After the perfusion, the spinal segments T10 was removed and postfixed in the same fixative for 2-4 h and then cryoprotected for 24 h at 4°C in 0.1 M PB containing 30% sucrose.

Transverse spinal sections (30 μm thickness) were cut in a cryostat, collected in 0.01 M phosphate-buffered saline (PBS, pH 7.3) and were then processed for immunofluorescent staining. Sections were rinsed in 0.01 M PBS (pH 7.3) for three times (10 min each), and then blocked with 2% goat serum in 0.01 M PBS containing 0.3% Triton X-100 for 1 h at the room temperature (RT) and then used for immunofluorescent staining. The sections were incubated overnight at 4°C with the primary antibodies: rabbit anti-TLR 3 (1: 100; Santa-Cruz Biotechnology) mixed with mouse anti- GFAP (1: 5000; Millipore, Temecula, CA, USA) or mouse anti-neuronal-specific nuclear protein (NeuN, 1:5000; Millipore) or mouse anti-OX-42 (1: 1000; Millipore). The sections were then washed for three times in 0.01 M PBS (10 min each) and then incubated for 2 h at RT with the corresponding secondary antibody: FITC-conjugated horse anti-mouse IgG (1:200; Vector, Burlingame, CA, USA) and Alexa 594-conjugated donkey anti-rabbit IgG (1:800; Molecular Probes, Rockford, IL, USA). Images were obtained using a confocal laser microscope (FV1000; Olympus, Tokyo, Japan) and digital images were captured with Fluoview 1000 (Olympus). Twelve nonadjacent sections were selected randomly from all the sections for the scanning. The z-separation was 4.6 μm under 20 × objective magnifications and was 1.0 μm under 60 × objective magnifications.

### Data analysis

All data were collected by experimenters blind to the surgery and treatments and statistical analysis was done by using SPSS software (version 12.0). Data were expressed as means ± standard error mean (means ± S.E.M.). Repeated measures ANOVA (with Bonferroni confidence interval adjustment) was used and conducted for data of pancreatic pathology. Two-way analysis of variance (ANOVA) (Group × Day) was used for analysis of behavioral data as well as Western blot and real-time PCR analysis. Dunnett's C *post hoc *test, assuming the variances were not equal, was employed whenever appropriate and significance was set at 0.05 for all statistical tests.

## Abbreviations

ASO: antisense oligodeoxynucleotide; COX-2: cyclooxygenase-2; DRG: dorsal root ganglia; ERK: extracellular signal-regulated kinase; GFAP: glial fibrillary acidic protein; IL: interleukin; JNK: c-Jun N-Terminal Kinase; MCP-1: monocyte chemotactic protein1; MO: oligodeoxynucleotide; PB: phosphate buffer; PBS: phosphate buffered saline; RF: response frequencies; RT: room temperature; RT-PCR: reverse transcription polymerase chain reaction; T: thoracic; TNBS: trinitrobenzene sulfonic acid; TLR: toll like receptor; TNF: tumor necrosis factor.

## Competing interests

The authors declare that they have no competing interests.

## Authors' contributions

NSQ and YHL performed the animal surgery, Western blot, and immunofluorescence and drafted the manuscript. QXF performed the behavioral test. YT performed the statistical analysis. KFD and KST conceived of the study, and participated in its design and coordination. All authors read and approved the final manuscript.
